# Sustained Elevated Cytokine Levels during Recovery Phase of Mayaro Virus Infection

**DOI:** 10.3201/eid2204.151502

**Published:** 2016-04

**Authors:** Dennis Tappe, José Vicente Pérez-Girón, Gudrun Just-Nübling, Gernot Schuster, Sergio Gómez-Medina, Stephan Günther, César Muñoz-Fontela, Jonas Schmidt-Chanasit

**Affiliations:** Bernhard Nocht Institute for Tropical Medicine, Hamburg, Germany (D. Tappe, S. Günther, C. Muñoz-Fontela, J. Schmidt-Chanasit);; German Centre for Infection Research, Hamburg (D. Tappe, S. Günther, C. Muñoz-Fontela, J. Schmidt-Chanasit);; Leibniz Institute for Experimental Virology, Hamburg (J. Vicente Pérez-Girón, S. Gómez-Medina, C. Muñoz-Fontela);; J.W. Goethe University Center for Infectious and Tropical Diseases, Frankfurt, Germany (G. Just-Nübling);; Praxis Schuster, Reinhardshagen, Germany (G. Schuster)

**Keywords:** Mayaro virus, arthralgia, cytokine, inflammation, alphavirus, travel, imported infection, arbovirus, viruses

**To the Editor:** Mayaro virus (MAYV), a mosquitoborne alphavirus endemic to South America, causes a self-limiting febrile arthralgia syndrome closely resembling Chikungunya fever (*1*). MAYV has been detected increasingly as imported infections in international travelers returning to Europe and North America (*2*–*9*). Joint pain, the most prominent symptom, is often long-lasting (several months), sometimes incapacitating (*4*,*6*,*7*,*9*), and may recur (*8*). Arthralgia develops during the acute phase and symmetrically affects the wrists, ankles, and small joints of hands and feet. Joint swelling may occur initially, but permanent joint damage has not been described (*5*). The clinical disease and diagnostic procedures have been described (*1*–*9*), but immunologic parameters and their possible role in the clinical follow-up of patients (i.e., during the postacute long-lasting arthralgia period) remain to be investigated.

To further our knowledge of MAYV infection, we analyzed cytokine levels in serum samples from 6 travelers to South America who returned to Europe with Mayaro fever (MF). Two of the cases occurred during 2014; 4 occurred during 2011–2013 (*2**–**5*).

The 6 travelers comprised 2 men and 4 women who were 20–54 (median 36) years of age (Technical Appendix Table). The 2 most recent cases occurred in spring 2014 in a 28-year-old female student and a 54-year-old male physician. Serologic testing was performed for both patients at the Bernhard Nocht Institute and confirmed by virus neutralization testing (*4*).

The student had traveled for 3 weeks in Ecuador, visiting rainforest villages and hiking in the jungle. During her stay, she had experienced myalgia of the forearms, arthralgia of fingers and toes, subfebrile but elevated body temperatures, and maculopapular exanthema. On examination in Germany, the student had no clinical signs of disease, but she reported arthralgia of the ankles and hands. Laboratory test results showed a slightly increased C-reactive protein level (6.2 mg/L, reference value <5 mg/L); liver and kidney values and blood count were within reference ranges. MAYV indirect immunofluorescence assay showed positive IgM and IgG titers (1:320 and 1:2,560, respectively; cut-off for both was <1:20) (*3*). Acute MF was diagnosed. Two weeks later, follow-up serologic testing showed negative IgM but unchanged IgG titers. Arthralgia with stiffness lasted for 6 weeks.

The physician had traveled for 3 weeks through the jungle in Bolivia, during which time headache, myalgia, shivers, and fatigue developed, followed by foot arthralgia and maculopapular exanthema. On his return to Germany, he was seen in an outpatient practice for persisting (2 months) bilateral foot pain. Laboratory test results showed C-reactive protein levels, and liver and kidney function values, and full blood count within reference ranges. MAYV indirect immunofluorescence assay was negative for IgM but positive for IgG (1:20,480). Postacute MAYV infection was diagnosed. Two months later, follow-up serologic testing showed unchanged titers. Arthralgia with pronounced morning stiffness lasted for 6 months.

After obtaining written consent from all patients, we subjected their serum samples to multiplex cytokine serum analyses (Bio-Rad Laboratories, Munich, Germany). Blood was drawn at different times after symptom onset (15–117 days). Serum samples were classified as acute (<30 days after symptom onset, n = 3) or postacute (>30 days after symptom onset, with arthralgia, n = 8). Twenty serum samples from healthy blood donors were run in parallel. During the acute phase of MF, interleukin (IL) 10, IL-12p70, RANTES (regulated on activation, normal T cell expressed and secreted), and vascular endothelial growth factor concentrations for patients were significantly elevated compared with those for healthy controls (Figure). Furthermore, a significant decrease was noted for eotaxin levels during the acute phase of disease. In the postacute arthralgic recovery phase, concentrations of IL-5–10, IL-13, IL-17, IP-10 (interferon-γ–induced protein 10), RANTES, macrophage inflammatory proteins 1α and 1β, granulocyte-macrophage colony-stimulating factor, and interferon-γ were significantly higher than those for healthy controls. TNF-α concentrations showed a nonsignificant median decrease during the acute phase (Figure). No significant changes in either phase were demonstrated for IL-1b, IL-2, IL-4, basic fibroblast growth factor, granulocyte colony-stimulating factor, monocyte chemotactic protein 1, and platelet-derived growth factor β polypeptide levels (data not shown). Cytokine levels measured in the acute phase did not differ significantly from those measured in the recovery phase.

**Figure:**
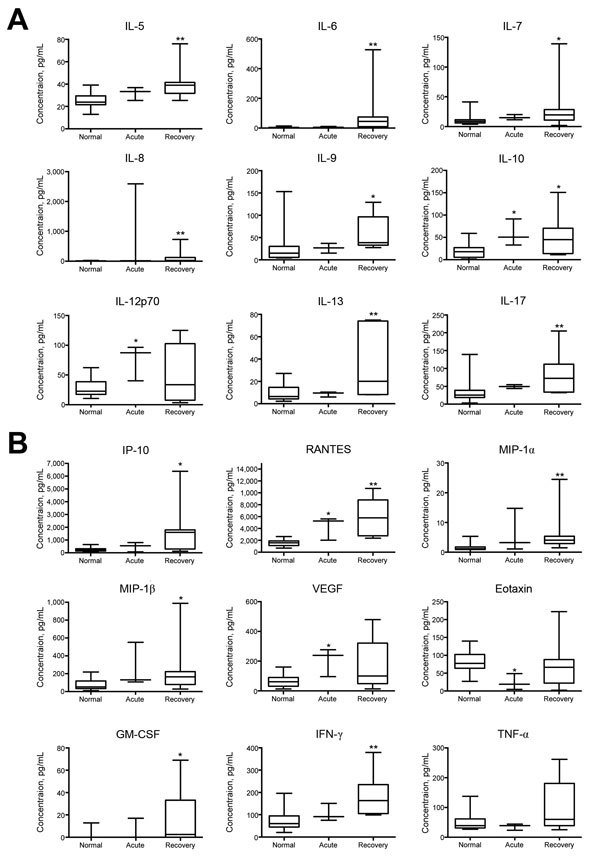
Changes in cytokine and growth factor levels in the acute and recovery phase of Mayaro fever. Box-and-whisker plots show median, upper and lower quartile, minimum, and maximum values. A) During the prolonged recovery phase, serum levels of interleukin (IL) 5–10, IL-13, and IL-17 were significantly elevated compared with levels for healthy controls. IL-10 levels were also significantly increased during the acute phase, as were IL-12p70 levels. B) Significantly increased serum concentrations of interferon-γ (IFN-γ)–induced protein 10 (IP-10), regulated on activation, normal T cell expressed and secreted (RANTES), macrophage inflammatory proteins (MIP)–1α and –1β, granulocyte-macrophage colony-stimulating factor (GM-CSF), and IFN-γ were detected in the prolonged recovery phase; significant elevations were also seen in the acute phase for RANTES and vascular endothelial growth factor (VEGF). Tumor necrosis factor α (TNF-α) concentrations showed a nonsignificant median decrease during the acute phase, whereas eotaxin levels were significantly decreased at that time. *p<0.05 and **p<0.01, versus values for healthy controls (Kruskal-Wallis test).

The most striking clinical feature of MF is long-lasting arthralgia, similar to that seen in chikungunya fever, which develops in the acute phase and persists thereafter. In the travel-associated cases, arthralgia lasted for 2 to >12 months. In the examined patients, the prolonged arthralgia recovery phase was paralleled by significantly increased proinflammatory cytokine levels, indicating ongoing inflammation, probably related to arthritis. Elevated levels of RANTES and IP-10 suggest T-cell recruitment, possibly reflecting virus persistence and replication, as described for the related Chikungunya virus (*10*). Thus, cytokine measurements may be helpful for monitoring patient symptoms, especially when signs of arthritis (swellings and redness) and elevated standard serum inflammatory parameters are no longer present. In our study, the picture of MF cytokine elevations paralleled those described for Chikungunya virus infection (*10*); these elevated levels may help to elucidate the pathogenesis of MAYV-induced arthralgia. More immunology data are required to complete this evolving picture of viral arthralgia syndromes.

Technical Appendix International travelers who were recently diagnosed with Mayaro fever and were included in this study.
