# Supplementary material for: Sustained Elevated Cytokine Levels during Recovery Phase of Mayaro Virus Infection
Source: Emerg Infect Dis. 2016 Apr;22(4):750–2. doi: 10.3201/eid2204.151502 (PMC4806971; doi:10.3201/eid2204.151502)
Supplement: Technical Appendix — International travelers who were recently diagnosed with Mayaro fever and were included in this study. [file 15-1502-Techapp-s1.pdf]

Article DOI: http://dx.doi.org/10.3201/eid2204.151502

Sustained Elevated Cytokine Levels during Recovery Phase of Mayaro Virus Infection

Technical Appendix

Technical Appendix Table. International travelers recently diagnosed with Mayaro fever included in the study.

Case No.	Year of Infection	Country of Infection	Imported to	Sex	Age, y	Risk Activity (Locality)	Laboratory diagnosis	Reference	Cytokine level testing, days after disease onset
1	2014	Ecuador	Germany	F	28	Jungle Trekking (Macas, Puyo, Tena villages)	Serology	This report	26 and 45
2	2014	Bolivia	Germany	M	54	Jungle Trekking (Buenavista village)	Serology	This report	74, 88, and 117
3	2013	Brazil	The Netherlands	F	52	Research in the Rainforest (Caxiuana village)	Serology	(*1*)	15
4	2013	French Guiana	Germany	F	44	Butterfly Hunting (St. Laurent, Cayenne, Cacao Cities)	PCR and Serology	(*2*)	16 and 33
5	2012	Bolivia	Germany	F	20	Work in Wildlife Resort (Rurrenabaque Region)	Serology	(*3*)	83 and 106
6	2011	Peru	Switzerland	M	27	Vacation in Small Rainforest City (Tarapoto village)	Serology	(*4*)	44

References

1. Slegers CA, Keuter M, Günther S, Schmidt-Chanasit J, van der Ven AJ, de Mast Q. Persisting arthralgia due to Mayaro virus infection in a traveler from Brazil: is there a risk for attendants to the 2014 FIFA World Cup? J Clin Virol. 2014;60:317–9. PubMed http://dx.doi.org/10.1016/j.jcv.2014.04.020

2. Friedrich-Jänicke B, Emmerich P, Tappe D, Günther S, Cadar D, Schmidt-Chanasit J. Genome analysis of Mayaro virus imported to Germany from French Guiana. Emerg Infect Dis. 2014;20:1255–7. PubMed http://dx.doi.org/10.3201/eid2007.140043

3. Theilacker C, Held J, Allering L, Emmerich P, Schmidt-Chanasit J, Kern WV, et al. Prolonged polyarthralgia in a German traveller with Mayaro virus infection without inflammatory correlates. BMC Infect Dis. 2013;13:369. PubMed http://dx.doi.org/10.1186/1471-2334-13-369

4. Neumayr A, Gabriel M, Fritz J, Günther S, Hatz C, Schmidt-Chanasit J, et al. Mayaro virus infection in traveler returning from Amazon Basin, northern Peru. Emerg Infect Dis. 2012;18:695–6. PubMed http://dx.doi.org/10.3201/eid1804.111717
